# The *c.470 T > C CHEK2* missense variant increases the risk of differentiated thyroid carcinoma in the Great Poland population

**DOI:** 10.1186/s13053-015-0030-5

**Published:** 2015-03-01

**Authors:** Marta Kaczmarek-Ryś, Katarzyna Ziemnicka, Szymon T Hryhorowicz, Katarzyna Górczak, Justyna Hoppe-Gołębiewska, Marzena Skrzypczak-Zielińska, Michalina Tomys, Monika Gołąb, Malgorzata Szkudlarek, Bartłomiej Budny, Idzi Siatkowski, Paweł Gut, Marek Ruchała, Ryszard Słomski, Andrzej Pławski

**Affiliations:** 1 Institute of Human Genetics, Polish Academy of Sciences, Ul. Strzeszyńska 32, Poznań, 60-479 Poland; 2 Department of Endocrinology, Metabolism and Internal Diseases, University of Medical Sciences, Poznań, Poland; 3 Department of Mathematical and Statistical Methods, University of Life Sciences, Poznań, Poland; 4 Institute for Applied Human Genetics and Oncogenetics, Zwenkau, Germany; 5 Department of Biochemistry and Biotechnology, University of Life Sciences, Poznań, Poland

**Keywords:** Differentiated thyroid carcinoma, CHEK2 gene sequence variants, Genotyping, Cancer risk factors

## Abstract

**Background:**

Differentiated thyroid carcinoma (DTC) originates from thyroid follicular epithelial cells and belongs to a group of slowly progressing tumors with a relatively good prognosis. However, recurrences and metastases are a serious problem in advanced stages. Furthermore, progression from a well differentiated thyroid carcinoma to an aggressive anaplastic one is possible.

The majority of differentiated thyroid carcinomas are sporadic but a few alleles increasing the cancer risk are known. One of them is the c.470 T > C (p.I157T, rs17879961) missense substitution in the *CHEK2* gene.

**Aim of the study:**

The aim of this study was to investigate whether this specific *CHEK2* alteration, c.470 T > C, predisposes the Great Poland (Wielkopolska) population to thyroid cancer.

**Methods:**

602 differentiated thyroid carcinoma patients and 829 controls randomly selected from population were genotyped for the presence of the c.470C allele using pyrosequencing. Hardy-Weinberg Equilibrium (HWE) was tested for both groups by chi-square distribution and Fisher’s exact test. The odds ratios (ORs), 95% confidence intervals (CIs), and p-values were calculated using the R software.

**Results:**

The results of genotyping showed the presence of the c.470C allele in 51 patients with a frequency of 4.49%, while in a controls in 42 patients with a frequency of 2.53%. We demonstrated that in the Great Poland population the c.470C *CHEK2* variant increases the risk of developing differentiated thyroid cancer almost twice (OR = 1.81, p = 0.004). The risk of papillary thyroid carcinoma in female patients homozygous for the c.470C allele was shown to increase almost 13-fold (OR = 12.81, p = 0.019).

**Conclusions:**

Identification of c.470C *CHEK2* gene variant ought to be taken into account by healthcare policymakers. Future well-designed and larger population studies are of great value in confirming these findings. Moreover, a combination of genetic factors together with environmental exposures should also be considered.

**Electronic supplementary material:**

The online version of this article (doi:10.1186/s13053-015-0030-5) contains supplementary material, which is available to authorized users.

## Introduction

Differentiated thyroid carcinoma (DTC) originates from thyroid follicular cells and is the most common malignancy of the endocrine system. In the Polish population, it accounts for almost 1.5% of all cancers. The most frequently occuring differentiated thyroid carcinoma types are papillary thyroid carcinoma (PTC; 65-90% of cases), follicular carcinoma (FTC; 10%-35%), and Hurthle cell or oxyphilic tumors (5%). More than 2,000 new cases of differentiated thyroid carcinoma are reported in Poland each year (2389 cases in 2011) [1]. The majority of patients are women. Generally, in various populations thyroid cancer occurs approximately four to six times less frequently in males, which indicates different molecular backgrounds between the two genders [2].

The etiology of thyroid cancer is not well understood except for a few percent of cases that are inherited or are a part of some cancer syndromes. However, in most cases thyroid cancer results from an interaction of several environmental (chemical agents, irradiation, iodine deficiency) and genetic factors [3]. Iodine intake plays a particular role in the pathogenesis of thyroid cancer. The papillary thyroid carcinoma is more often observed in areas of sufficient iodine diet content, while follicular thyroid carcinoma appears more frequently in areas of iodine deficiency. In Poland, during the fifteen years following the introduction of iodine prophylaxis, the follicular thyroid carcinoma frequency has decreased, whereas the incidence of papillary thyroid carcinoma and lymphocytic infiltration due to autoimmune thyroiditis has increased significantly [4].

Most studies show that papillary thyroid carcinoma and follicular thyroid carcinoma patients have similar prognoses [5], but some authors reported significant differences in the behavior of follicular and papillary thyroid carcinomas, even though they are derived from the same follicle cells [6]. Papillary thyroid carcinomas tend to grow very slowly and usually develop in only one lobe of the thyroid gland. They often spread to the lymph nodes in the neck but still have a good prognosis. Follicular thyroid carcinomas usually metastasize to distant tissues: lungs or bones. In advanced stages, they do not give such a favorable prognosis as papillary thyroid carcinomas. For unclear reasons, certain histological subtypes of papillary thyroid carcinoma may have a poor prognosis, similar to that of highly invasive forms of follicular carcinoma [7],[8].

The clinical evaluation of thyroid nodules is based on a detailed medical history, a physical examination and laboratory tests. Ultrasound examination with fine needle aspiration biopsy (FNAB) may improve the accuracy of cytological diagnosis and patient management [9], however, 30% of diagnoses using these methods remain inconclusive [10]. Researchers underline the necessity to identify and validate novel markers of increased thyroid cancer susceptibility [11]. Multiple studies on low penetrance cancer susceptibility alleles showed that alterations in the *CHEK2* gene increase the risk of different malignancies [12].

The *CHEK2* gene (22q12.1) encodes the human orthologue of the yeast checkpoint kinases Cds1 and Rad53. The CHEK2 protein is activated by phosphorylation in response to DNA damage and it inhibits the CDC25C phosphatase, thus preventing mitosis. It stabilizes the p53 tumor suppressor protein, which induces the arrest of the cell cycle in the G1 phase [13]. Falck and collaborators suggested that the ATM-CHEK2-CDC25A-CDK2 pathway is a genomic checkpoint that prevents the synthesis of DNA damaged by ionizing radiation [14]. In the early stages of tumorigenesis, human cells activate an ATR/ATM-regulated DNA damage response pathway, which delays the progression of cancer. Mutations in the ATM-CHEK2-p53 pathway lead to uncontrolled cell proliferation, high genomic instability, tumor cell evasion from apoptosis, and tumor progression [15]. It has been shown that four alterations in the *CHEK2* gene, 1100delC, IVS2 + 1G > A, del5395, and c.470 T > C predispose the Polish population to various cancers [16]. The most frequent of these is missense change c.470 T > C (p.I157T, rs17879961) with an incidence of 5 per 100 persons in the European populations [12],[16],[17]. This alteration is localized in a region coding for a functionally important FHA domain of the CHEK2 protein. The protein with a threonine in the 157 position is defective in its ability to bind p53, BRCA1 and Cdc25A proteins [14],[18],[19]. This altered CHEK2 protein may also have a dominant negative effect by forming heterodimers with the wild-type form [17].

The aim of this study was to determine whether the c.470 T > C (p.I157T, rs17879961) *CHEK2* allele increases the susceptibility to differentiated thyroid carcinoma in the Great Poland population.

## Materials and methods

### Patients and controls

We analyzed material from 602 patients with differentiated thyroid carcinoma (527 females and 75 males) from the Great Poland region. All of them were diagnosed at the Department of Endocrinology, Metabolism, and Internal Diseases of the Poznan University of Medical Sciences. The group consisted of 535 patients diagnosed with papillary thyroid carcinoma and 67 patients with follicular thyroid carcinoma. In the examined group we did not record family history of thyroid cancers, however we noticed two familial cases of papillary thyroid carcinoma. Furthermore in 26 patients we observed also other primary cancers (Table 1).

**Table 1:** The occurrence of other primary cancers in subjected patients with thyroid cancer

No.	Age	Gender	Histopathology	Other primary cancers	*CHEK2*c.470T>C
1	64	female	papillary cancer	breast cancer	TT
2	76	female	papillary cancer	breast cancer	TT
3	65	female	follicular cancer	breast cancer	**TC**
4	73	female	papillary cancer	breast cancer	**TC**
5	67	female	papillary cancer	breast cancer	TT
6	73	female	papillary cancer	breast cancer	TT
7	56	female	papillary cancer	breast cancer	TT
8	84	female	papillary cancer	breast cancer	TT
9	62	female	papillary cancer	breast cancer	TT
10	55	female	papillary cancer	endometrial cancer	TT
11	82	female	papillary cancer	endometrial cancer	TT
12	77	female	papillary cancer	basal cell skin carcinoma	TT
13	59	female	papillary cancer	uterine cervix carcinoma	TT
14	73	female	follicular cancer	mediastinal tumor	TT
15	28	female	papillary cancer	lymphoblastic lymphoma	**TC**
16	74	female	papillary cancer	renal cancer	TT
17	56	female	papillary cancer	endometrial cancer	TT
18	75	female	papillary cancer	squamous cell tongue carcinoma	**TC**
19	54	female	papillary cancer	colon carcinoma	TT
20	68	female	papillary cancer	uterine cervix carcinoma	**TC**
21	68	female	papillary cancer	lung tumor	TT
22	79	female	papillary cancer	vaginal carcinoma	TT
23	69	male	papillary cancer	malignant lymphoma	TT
24	67	male	papillary cancer	lung carcinoma	TT
25	32	male	papillary cancer	salivary gland carcinoma and follicular thyroid carcinoma	TT
26	37	male	papillary cancer	Non-Hodgkin's lymphoma	TT

The research material was collected between 2008 and 2012, and the majority of patients were newly diagnosed. The first patients were diagnosed in 2002 and are still alive. The date of disease onset was set as the date of the thyroidectomy. Information concerning histopathology was obtained from three major clinical centers in Poznan: Greater Poland Cancer Centre, the Regional Hospital in Poznan and Heliodor Swiecicki Clinical Hospital, where patients underwent surgery.

Clinical files were established at diagnosis and included age, histopathological examination results, and the TNM staging (Table 2).

**Table 2:** Clinical characteristics of thyroid cancer patients

Age groups (range)	Number of patients	%
0 - 30	80	15
30 - 40	98	18,5
40 – 50	115	21,6
50 - 60	133	25
60 -100	105	19,9
Staging		
pT1	258	48,3
pT2	102	19,1
pT3	75	14
pT4	70	13,2
pTx	29	5,4
Lymph node status		
N1	86	16
N0	451	84
Nx	0	0
Distant metastases		
M1	25	4,6
M0	512	95,4
Mx	0	0

The control group consisted of 829 unrelated individuals (562 females and 267 males) randomly chosen from adult members of families attending diagnostic laboratories in the Great Poland region for paternity testing. The number of female controls was doubled as compared to the number of females in the general population in order to match the male-to-female ratio in the group of patients with differentiated thyroid carcinoma.

All individuals agreed to take part in genetic testing and the study was approved by the local Ethical Committee of the Poznań University of Medical Sciences (approval no. 629/07).

### DNA extraction and genotyping

DNA was extracted from whole blood leukocytes using guanidine isothiocyanate and phenol-chloroform. Isolates were dissolved in 1xTE buffer and stored at −20°C until use. The *CHEK2* c.470 T > C (rs17879961) genotyping was performed by pyrosequencing. The sequences of the primers used in the analysis were designed by PyroMark Assay Design Software 2.0 (Biotage). The following primers were used: forward 5’-GCTGGTAATTTGGTCATTGTTTT, reverse biotinylated 5’-CATTGCCACTGTGATCTTCTAT (PCR product length 142 bp, Tm = 57°C), and sequencing primer 5’-TGGGTCCTAAAAACTCTT. Pyrosequencing reactions were performed on the PSQ96 apparatus using PyroMark Gold Q96 reagent kits (Qiagen) according to the manufacturer’s protocol.

### Statistical analysis

Statistical analysis included crude analysis for entire groups and calculations were adjusted by gender, histological cancer type and age at disease onset. Hardy-Weinberg Equilibrium (HWE) was checked for the c.470 T > C (p.I157T, rs17879961) variant in papillary thyroid carcinoma and follicular thyroid carcinoma patients, and in control subjects by chi-square distribution and Fisher’s exact test. The association between the c.470 T > C variant and differentiated thyroid carcinoma occurrence was estimated by calculating the odds ratios (ORs), 95% confidence intervals (CIs), and p-values using contingency tables. The independence between the two variables was verified using Pearson's chi-square test and Fisher’s exact test. To assess differences in the probabilities of the occurrence of specific variable level combinations, the null hypothesis was tested against a two-sided alternative hypothesis. Statistical significance was set at p < 0.05. All statistical calculations were performed using the R software v. 3.0.1 [20], a free software environment for statistical computing and graphics. As the CC genotype was not present in the studied population group, statistical amendment was applied (Review Manager 5) [21].

## Results and discussion

**Figure 1:**
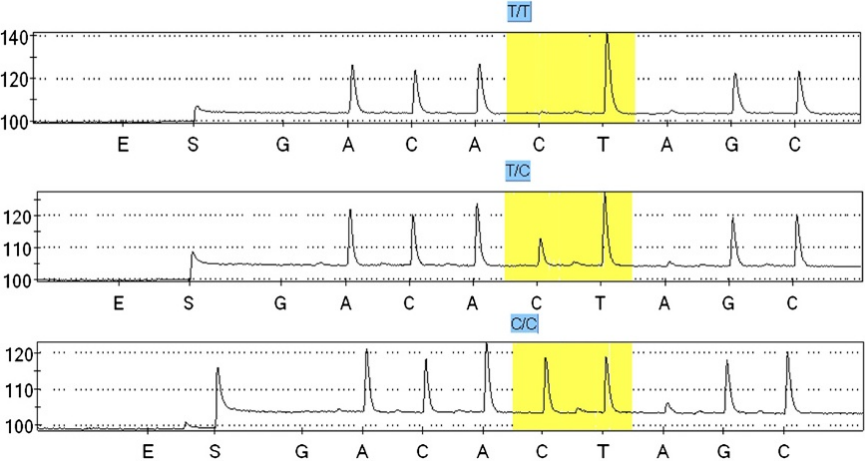
**Pyrosequencing profiles of the three genotypes of the c.470 T > C (p.I157T, rs17879961)** ***CHEK2*** **missense substitution.** Polymorphic site is marked in yellow.

In molecular analysis of fine needle aspiration biopsy samples, four well known mutant genes have been identified in papillary and follicular thyroid carcinoma: *BRAF*, *RAS*, *RET/PTC*, and *PAX8/PPARG*[22]. However, while it is possible to identify mutations in DNA from tumor cells, there is no clear information on other genetic factors associated with a predisposition to or initiation of a malignant thyroid transformation. We decided to analyze the frequency of the *CHEK2* c.470C allele in a cohort of 602 Polish differentiated thyroid carcinoma (DTC) cases and 829 controls using a rapid diagnostic test based on pyrosequencing technology. It allowed identification of three possible genotypes: TT, TC and CC (Figure 1).

Our results indicate that the c.470C allele is almost twice as frequent in thyroid carcinoma patients than in controls. The analysis of the allele c.470C frequency by histological type of tumor revealed statistically relevant differences between patients with papillary thyroid carcinoma and controls.

Allele c.470C occurred 47 times in 44 patients diagnosed with papillary thyroid tumors (4.39%), giving the odds ratio of 1.77 (CI = [1.15-2.70], p = 0.008). In a group of patients with follicular histology allele c.470C occurred in 7 heterozygotic carriers (5.22%) – the calculated odds ratio is 2.1 [CI = 0.93-4.82], but the latter result is not statistically significant (p = 0.096) (Table 3).

**Table 3:** The occurrence of the c.470 T > C*CHEK2*gene variant in Polish differentiated thyroid carcinoma (DTC) patients and controls

		Group size	Genotypes			p-value	OR (95%CI)	Allele number			p-value	OR (95%CI)
			TT	TC	CC	Allele positivity (TT vs TC+CC)		Total	T	C	Allele frequency	
**DTC patients:**	All	602	551 (91,53%)	48 (7,97%)	3 (0,5%)	**0.009**	**1.73** (1.14-2.65)	1204	1150 (95,51%)	54 (4,49%)	**0.004**	**1.81** (1.20-2.72)
By cancer type:	PTC	535 (88,87%)	491 (91,78%)	41 (7,66%)	3 (0,56%)	**0.019**	**1.68** (1.08-2.60)	1070	1023 (95,61%)	47 (4,39%)	**0.008**	**1.77** (1.15-2.70)
	FTC	67 (11,13%)	60 (89,55%)	7 (10,45%)	n.o.	0.062	2.19 (0.94-5.08)	134	127 (94,78%)	7 (5,22%)	0.096	2.12 (0.93-4.82)
By gender:	DTC females	527 (87,54%)	479 (90,89%)	45 (8,54%)	3 (0,57%)	**0.017**	**1.72** (1.08-2.74)	1082	1031 (95,29%)	51 (4,71%)	**0.001**	**1.80** (1.14-2.82)
	DTC males	75 (12,46%)	72 (96%)	3 (4%)	n.o.	0.963	0.97 (0.26-3.57)	156	153 (98,08%)	3 (1,92%)	1.009	0.97 (0.27-3.52)
**Controls**	All	829	787 (94,93%)	42 (5,07%)	n.o.	-	-	1658	1616 (97,47%)	42 (2,53%)	-	-
By gender:	Females	562 (67,8%)	531 (94,48%)	31 (5,52%)	n.o.	-	-	1124	1093 (97,24%)	31 (2,76%)	-	-
	Males	267 (32,2%)	256 (95,88%)	11 (4,12%)	n.o.	-	-	534	523 (97,94%)	11 (2,06%)	-	-

In bold: statistically significant numbers with 95% confidence intervals.

n.o. not observed.

In an analysis by gender, we observed a strong association of the c.470C allele frequency with thyroid carcinoma risk in women with odds ratio of 1.8 (p = 0.001). The female carriers of this allele (CC or CT genotype) had OR = 1.7 (p = 0.017) for thyroid cancer (Table 3). Being a rare CC homozygote increased the risk of papillary thyroid carcinoma ten-fold (OR = 10.0, C.I. = [0.52-193.90], p = 0.039). Moreover, a rare homozygous genotype CC was detected only in 3 women with papillary thyroid carcinoma (0.57%) and it increased the risk of this cancer type almost thirteen times compared to that of the general population group (OR = 12.81, C.I. = [0.66-248.46], p = 0.019).

Our present data do not show any association between *CHEK2* gene variant c.470 T > C and the presence of DTC in male subjects. However, it may be the result of lower frequency of differentiated thyroid cancers in males and it may be related to the small sample size (75 male patients). Studies including larger sample size would be justified.

Clinical data concerning age at disease onset were available for 531 patients. We divided the differentiated thyroid carcinoma patients into five age groups and investigated the dependence between the age at disease onset as a categorical variable and the presence of the c.470C allele. The chi-square statistic was 9.76, which compared to a χ2 distribution with four degrees of freedom, gives a p-value of 0.045. We found a relationship between the presence of the c.407C genotype and age of disease onset. The c.407C genotype was more frequent in differentiated thyroid carcinoma patients diagnosed between the age of 51 and 60 (OR = 2.2, C.I. = [1.07-4.38]), and these results were statistically significant (p = 0.016; Table 4).

**Table 4:** CHEK2 variant c.470C occurrence in patients with differentiated thyroid carcinoma (DTC) in the age groups due to the age at onset

age group (years)	Number of patients	Number of patients with genotypes TC+CC (frequency)	OR	CI	p-value
0 - 30	80	7 (8.75%)	1.1	[0.41-2.74]	0.763
30 - 40	98	8 (8.16%)	1.0	[0.40-2.40]	0.918
40 – 50	115	8 (6.96%)	0.8	[0.33-1.92]	0.669
50 - 60	133	**17 (12.78%)**	**2.2**	**[1.07-4.38]**	**0.016**
60 -100	105	**2 (1.9%)**	**0.2**	**[0.02-0.75]**	**0.008**

In bold: statistically significant numbers with 95% confidence intervals.

An analysis of regional and distant metastases (as defined by the TNM classification) showed no statistically significant correlations with the presence of the c.407C allele (OR = 1.2, CI = [0.49-3.54], p = 0.694 for regional metastases, and OR = 1.1, CI = [0.24-9.31], p = 1 for distant metastases). The c.470C allele and multifocality occurred also as indepent traits (OR = 1, C.I. = [0.44-2.54], p = 0.997).

In differentiated thyroid carcinoma patients, the presence of the c.470C allele of the *CHEK2* gene increased the risk of developing the disease twice. A relationship of alterations in the *CHEK2* gene with different types of cancer have been previously reported by others [23]-[27]. Studies carried out in Finland have shown a relationship of the c.470C allele with an increased risk of breast cancer with an odds ratio of 1.4 [17]. Cybulski and collaborators detected the *CHEK2* c.470 T > C alteration at a frequency of 7.1% (OR = 1.5) in Polish female breast cancer patients. They found an even higher odds ratio for women diagnosed after fifty years of age [25].

Functionally defective variants of the *CHEK2* gene have been shown to be also linked with an increased predisposition to colon cancer [26],[28]. Three changes in this gene: IVS2 + 1G > A, 1100delC and c.470 T > C were previously reported as correlated with a higher risk of five types of cancer (breast, prostate, colon, thyroid, kidney) in the Polish population by Cybulski and collaborators. However, the thyroid cancer group analyzed in their study included only 173 patients from the North of Poland, which seems low given that this is a frequent cancer, and no data on histopathological types were provided [12].

To date *CHEK2* mutations have been associated with an increased risk of cancer at several different sites, including breast, prostate, thyroid, colon, kidney, stomach, (low-grade) ovarian, bladder, chronic lymphocytic leukemia, non-Hodgkin’s lymphoma, and a reduced risk of other cancers (lung and laryngeal cancer) [12],[29]-[36]. Variant *CHEK2* c.470 T > C was also associated with breast cancer in Poland, Finland, Germany and Belarus. The odds ratio for breast carcinoma given the I157T mutation was 1.5 in Poland, 1.4 for the Finnish population, 3.6 in the German population and 4.5 in the Byelorussian population [16],[17],[37]. Our results confirmed also recently published research from another region of Poland (central Poland) conducted on a large group of patients with papillary thyroid cancer, which showed OR = 2.2 (p = 2.37^−10^) [38].

In accordance with the results published in earlier reports, we demonstrated that differentiated thyroid carcinoma is more frequent in women. Among our patients, females were 7.5 times more likely to develop a papillary thyroid carcinoma than males. In a group of patients diagnosed with follicular carcinoma, the male to female ratio was 1:6. We hypothesized that, due to a higher risk of thyroid cancer among women, the occurrence of allele C may have a prognostic value. Actually, in our study, the presence of allele C indicated an almost two-fold increase in the differentiated thyroid carcinoma risk. A comparison of male differentiated thyroid carcinoma patients with men from the control group did not show statistically significant differences in the frequency of the alleles and genotypes of *CHEK2* gene.

Some earlier studies showed a correlation of this minor *CHEK2* allele c.470C with an early onset of breast cancer [39],[40]. We observed a different trend for thyroid cancer: the frequency of allele c.470C was increased more than two-fold in patients who were between 51 and 60 years of age at disease onset (OR = 2.2, C.I. = [1.07-4.38], p = 0.016). However, in patients who were diagnosed at over 60 years of age, the frequency of allele c.470C was lower with and odds ratio of 0.2 (CI = [0.02-0.75], p = 0.008) (Table 4). It should also be noted that the number of cases in this group is relatively small, so in order to draw a clear conclusion, an examination of a larger group in this age range should be performed.

The availability of clinical data allowed us to test a hypothesis that the rare c.470C allele of the *CHEK2* gene may be correlated with metastases. A statistical analysis showed that it is not a risk factor for regional or distant metastases as defined by the TNM classification. We did not observe any correlation of the presence of this allele with metastases (OR = 1.4, C.I. = [0.55-4.01], p = 0.496) or multifocality (OR = 1, C.I. = [0.44-2.54], p = 0.997).

**Figure 2:**
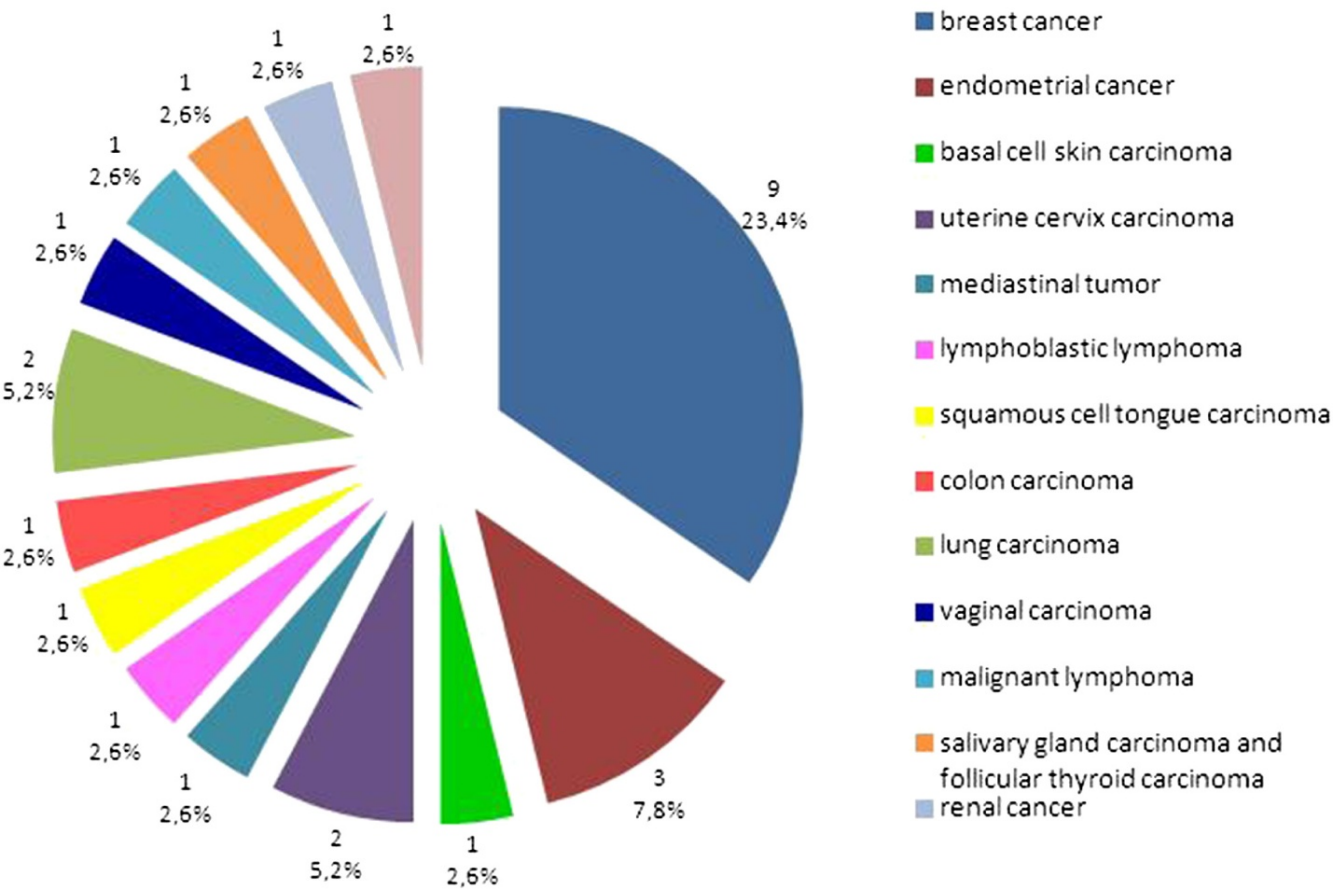
The occurrence of other primary cancers in patients with thyroid carcinoma.

Moreover, in 26 patients, we observed the coexistence of other primary cancers (Table 2, Figure 2). The most common co-morbid was breast cancer (9 patients, 23.4%). In three patients endometrial cancer was identified (7.8%). In this group of patients with second primary cancer, allele c.470C was present in 5 patients (19.2%).

We identified three rare homozygotes among our patients. Two of them were diagnosed with cancer at a young age (33 and 34 years), in stage T2. Moreover, one of those patients had metastases to the submandibular lymph nodes. In the third patient, the cancer was detected at the age of 59, in stage T3N1a. Certainly, it is impossible to reach an unambiguous conclusion on the impact of the homozygotic state on the disease course based on three cases but this is an interesting finding, which suggests that being a homozygote may predispose to young age thyroid cancer. Actually, the mean cancer onset age for these homozygotes was seven years lower than for all female patients in our study.

## Conclusions

We have demonstrated that the c.470 T > C substitution in the *CHEK2* gene is associated with an almost two-fold increase in the risk of papillary thyroid carcinoma in the Great Poland population, which confirms last published results for central Poland and earlier research on Pomeranian population. In homozygous women, it increases the risk of papillary thyroid carcinoma almost 13-fold. The presence of the c.470C allele significantly raises the risk of thyroid carcinoma in the midlife, but it is not a risk factor for the late occurrence of thyroid carcinoma. In our opinion, the carriers of the c.470 T > C alteration in the *CHEK2* gene, especially in the homozygous state, in addition to other cancers, are also at an increased risk of differentiated thyroid carcinoma. This should be taken into account by healthcare policymakers. Population screening for this *CHEK2* gene variant should be considered to be an effective strategy for differentiated thyroid carcinoma early detection and control.

## Authors’ original submitted files for images

Below are the links to the authors’ original submitted files for images. Authors’ original file for figure 1 Authors’ original file for figure 2

## References

[1] Wojciechowska U, Dzidkowska J, Zatoński W (2013). Nowotwory złośliwe w polsce w 2011 roku – krajowy rejestr nowotworów.

[2] Pellegriti G, Frasca F, Regalbuto C, Squatrito S, Vigneri R (2013). Worldwide increasing incidence of thyroid cancer: update on epidemiology and risk factors.

[3] Harach HR, Ceballos GA (2008). Thyroid cancer, thyroiditis and dietary iodine, a review based on the Salta, Argentina model. Endocr Pathol.

[4] Zdanowska-Filipczak J, Orlicz-Sczęsna G (2008). Epidemiological aspects of iodine deficit and excess. Zdr Publ.

[5] Hundahl SA, Fleming ID, Fremgen AM, Menck HR (1998). A national cancer data base report on 53,856 cases of thyroid carcinoma treated in the U.S., 1985–1995. Cancer.

[6] Volaru K, Norsaadach B, Naing NN, Biswal BM (2006). Prognostic factors of differentiated thyroid cancer patients in Hospital University Sains Malaysia. Singapore Med J.

[7] Lo CY, Chan WF, Lam KY, Wan KY (2005). Follicular thyroid carcinoma: the role of histology and staging systems in predicting survival. Ann Surg.

[8] D’Avanzo A, Treseler P, Ituarte PH, Wong M, Streja L, Greenspan FS (2004). Follicular thyroid carcinoma: histology and prognosis. Cancer.

[9] Hambleton C, Kandil E (2013). Appropriate and accurate diagnosis of thyroid nodules: a review of thyroid fine-needle aspiration. Int J Clin Exp Med.

[10] Gharib H, Papini E, Paschke R, Duick DS, Valcavi R, Hegedus L (2010). American association of clinical endocrinologists, associazione Medici endocrinologi, and European thyroid association medical guidelines for clinical practice for the diagnosis and management of thyroid nodules. Endocr Pract.

[11] Kunavisarut T (2013). Diagnostic biomarkers of differentiated thyroid cancer. Endocrine.

[12] Cybulski C, Górski B, Huzarski T, Masojć B, Mierzejewski M, Dębniak T (2004). CHEK2 is a multiorgan cancer susceptibility gene. Am J Hum Genet.

[13] Matsuoka S, Huang M, Elledge S (1998). Linkage of ATM to cell cycle regulation by the chek2 protein kinase. Science.

[14] Falck J, Lukas C, Protopopova M, Lukas J, Selinanova G, Bartek J (2001). Functional impact on concomitant versus alternative defects in the chek2-p53 tumour suppressor pathway. Oncogene.

[15] Bartkova J, Horejsi Z, Koed K, Kramer A, Tort F, Zieger K (2005). DNA damage response as a candidate anti-cancer barrier in early human tumorigenesis. Nature.

[16] Cybulski C, Huzarski T, Górski B, Masojć B, Mierzejewski M, Debniak T (2004). A novel founder CHEK2 mutation is associated with increased prostate cancer risk. Cancer Res.

[17] Kilpivaara O, Vahteristo P, Falck J, Syrjakoski K, Eerola H, Easton D (2004). CHEK2 variant I157T may be associated with increased breast cancer risk. Int J Cancer.

[18] Falck J, Mailand N, Syljuåsen RG, Bartek J, Lukas J (2001). The ATM-chek2-Cdc25A checkpoint pathway guards against radioresistant DNA synthesis. Nature.

[19] Li J, Williams BL, Haire LF, Goldberg M, Wilker E, Durocher D (2002). Structural and functional versatility of the FHA domain in DNA-damage signaling by the tumor suppressor kinase chek2. Mol Cell.

[20] R Development Core Team. A language and environment for statistical computing. R Foundation for Statistical Computing. 2014. [http://www. r-project.org].

[21] Deeks JJ, Higgins JPT (2010). Statistical algorithms in Review Manager 5.

[22] Eszlinger M, Krogdahl A, Muenz S, Rehfeld C, Precht Jensen E, Ferraz C (2014). Impact of molecular screening for point mutations and rearrangements in routine air-dried Fine Needle Aspiration samples of thyroid nodules. Thyroid.

[23] Dong X, Wang L, Taniguchi K, Wang X, Cunningham JM, McDonnell SK (2003). Mutations in CHEK2 associated with prostate cancer risk. Am J Hum Genet.

[24] Schutte M, Seal S, Barfoot R, Meijers-Heijboer H, Wasielewski M, Evans DG (2003). Variants in CHEK2 other than 1100delC Do Not make a major contribution to breast cancer susceptibility. Am J Hum Genet.

[25] Cybulski C, Wokołorczyk D, Jakubowska A, Huzarski T, Byrski T, Gronwald J (2011). Risk of breast cancer in women with a CHEK2 mutation with and without a family history of breast cancer. J Clin Oncol.

[26] Kilpivaara O, Alhopuro P, Vahteristo P, Aaltonen LA, Nevanlinna H (2006). CHEK2 I157T associates with familial and sporadic colorectal cancer.

[27] Desrichard A, Bidet Y, Uhrhammer N, Bignon YJ (2011). CHEK2 contribution to hereditary breast cancer in non-BRCA families.

[28] Liu W, Zhong B, Zhang Y, Choi G (2007). Mutation analysis of the checkpoint kinase 2 gene in colorectal cancer cell lines. Chinese Med.

[29] Brennan P, McKay J, Moore L, Zaridze D, Mukeria A, Szeszenia-Dabrowska N (2007). Uncommon CHEK2 mis-sense variant and reduced risk of tobacco-related cancers: case control study. Hum Mol Genet.

[30] Cybulski C, Masojc B, Oszutowska D, Jaworowska E, Grodzki T, Waloszczyk P (2008). Constitutional CHEK2 mutations are associated with a decreased risk of lung and laryngeal cancers. Carcinogenesis.

[31] Wang Y, McKay JD, Rafnar T, Wang Z, Timofeeva MN, Broderick P (2014). Rare variants of large effect in BRCA2 and CHEK2 affect risk of lung cancer. Nat Genet.

[32] Szymanska-Pasternak J, Szymanska A, Medrek K, Imyanitov EN, Cybulski C, Gorski B (2006). CHEK2 variants predispose to benign, borderline and low-grade invasive ovarian tumors. Gynecol Oncol.

[33] Złowocka E, Cybulski C, Górski B, Debniak T, Słojewski M, Wokołorczyk D (2008). Germline mutations in the CHEK2 kinase gene are associated with an increased risk of bladder cancer. Int J Cancer.

[34] Rudd MF, Sellick GS, Webb EL, Catovsky D, Houlston RS (2006). Variants in the ATMBRCA2-CHEK2 axis predispose to chronic lymphocytic leukemia. Blood.

[35] Havranek O, Spacek M, Hubacek P, Mocikova H, Markova J, Trneny M (2011). Alterations of CHEK2 forkhead-associated domain increase the risk of Hodgkin lymphoma. Neoplasma.

[36] Teodorczyk U, Cybulski C, Wokołorczyk D, Jakubowska A, Starzyńska T, Lawniczak M (2013). The risk of gastric cancer in carriers of CHEK2 mutations. Fam Cancer.

[37] Bogdanova N, Enssen-Dubrowinskaja N, Feshchenko S, Lazjuk GI, Rogov YI, Dammann O (2005). Association of two mutations in the CHEK2 gene with breast cancer. Int J Cancer.

[38] Wójcicka A, Czetwertyńska M, Świerniak M, Długosińska J, Maciąg M, Czajka A (2014). Variants in the ATM-CHEK2-BRCA1 axis determine genetic predisposition and clinical presentation of papillary thyroid carcinoma. Genes Chromosom Cancer.

[39] Friedrichsen DM, Malone KE, Doody DR, Daling JR, Ostrander EA (2004). Frequency of CHEK2 mutations in a population based, case–control study of breast cancer in young women. Breast Cancer Res.

[40] Liu C, Wang Y, Wang QS, Wang YJ (2013). The CHEK2 I157T variant and breast cancer susceptibility: a systematic review and meta-analysis. Asian Pacific J Cancer Prev.

