# Supplementary figures and images for: The c.470 T > C CHEK2 missense variant increases the risk of differentiated thyroid carcinoma in the Great Poland population
Source: Hered Cancer Clin Pract. 2015 Mar 1;13:8. doi: 10.1186/s13053-015-0030-5 (PMC4367841; doi:10.1186/s13053-015-0030-5)

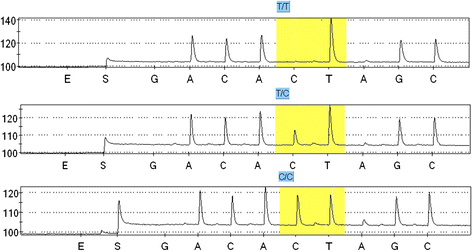

Supplement: Supplementary file 1 — Authors’ original file for figure 1 [file 13053_2015_30_MOESM1_ESM.gif]

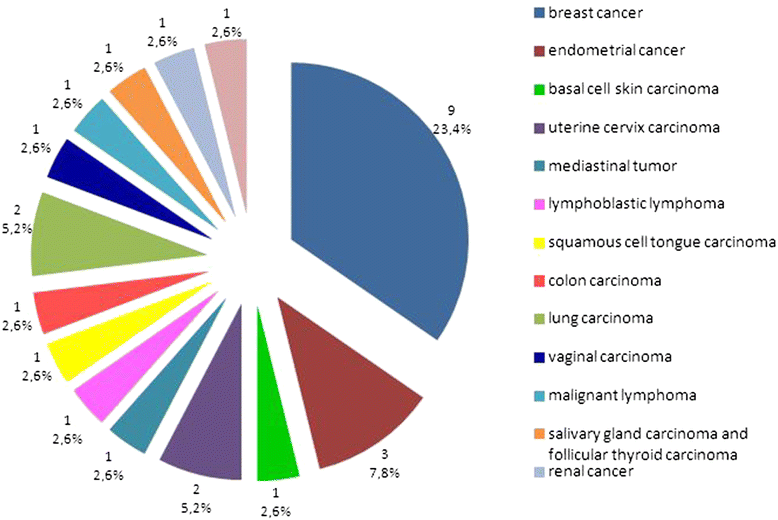

Supplement: Supplementary file 2 — Authors’ original file for figure 2 [file 13053_2015_30_MOESM2_ESM.gif]
